# Association of Common Variants in *OLA1* Gene with Preclinical Atherosclerosis

**DOI:** 10.3390/ijms231911511

**Published:** 2022-09-29

**Authors:** Ting-Fong Lin, Chao-Liang Chou, Chu-Jui Hsieh, Yih-Jer Wu, Yi-Cheng Chen, Tzu-Wei Wu, Shu-Xin Lu, Yue-Li Juang, Li-Yu Wang

**Affiliations:** 1 Institute of Biomedical Sciences, MacKay Medical College, New Taipei City 252005, Taiwan; 2 Department of Medicine, MacKay Medical College, New Taipei City 252005, Taiwan; 3 Department of Neurology, MacKay Memorial Hospital, New Taipei City 251404, Taiwan; 4 Cardiovascular Center, Department of Internal Medicine, Mackay Memorial Hospital, Taipei 104217, Taiwan

**Keywords:** atherosclerosis, carotid intima–media thickness, single nucleotide polymorphism, *BRCA1*, *OLA1*, genetic association study, population-based study

## Abstract

Reactive oxygen species impair the blood vessels, leading to the initiation of atherosclerosis, and migration and proliferation of vascular smooth muscle cells and neovascularization by endothelial cells of vasa vasorum are essential for atherosclerosis development. Obg-like ATPase 1 (OLA1), a negative regulator in cellular responses to oxidative stress, binds to breast cancer susceptibility gene 1 (BRCA1), which protects vascular endothelial and smooth muscle cells against reactive oxygen species. However, it is not known whether OLA1 is genetically correlated with atherosclerosis. Here, we conducted two independent population-based case–control studies to explore the effects of variants in *OLA1* genes on preclinical atherosclerosis. A total of 564 and 746 subjects who had thicker and normal carotid intima–media thickness (cIMT), respectively, were enrolled. Among 55 screened SNPs, rs35145102, rs201641962, rs12466587, rs4131583, and rs16862482 in *OLA1* showed significant associations with cIMT. SNP rs35145102 is a 3′-utr variant and correlates with the differential expression of OLA1 in immune cells. These five genetic markers form a single closely linked block and H1-ATTGT and H2-GCCTC were the top two most prevalent 5-locus haplotypes. The H1 + H1 genotype negatively and H1 + H2 genotype positively correlated with thicker cIMT. The five identified SNPs in the *OLA1* gene showed significant correlations with cIMT. Furthermore, we found that OLA1 was required for migration and proliferation of human aortic endothelial and smooth muscle cells and regulated vascular tube formation by human aortic endothelial cells. Therefore, these genetic variants in the *OLA1* gene may serve as markers for risk prediction of atherosclerotic diseases.

## 1. Introduction

Atherosclerosis is a multi-stage and multifactorial vascular disease. Three types of cells—immune cells, vascular endothelial cells (ECs), and vascular smooth muscle cells (SMCs)—play critical roles in the initiation and progression of athersclerosis and the stability of vascular plaque [1,2,3,4,5,6]. Early stages of atherosclerosis are related to blood flow disturbances that damage the endothelium, followed by platelet adhesion, macrophage penetration into the sub-endothelium, and migration and proliferation of vascular SMCs [1,2,3,4,5,6]. Later stages of atherosclerosis are characterized by the formation of unstable plaques, leading to thrombus formation and, potentially, embolism [1,2,3,4,5,6,7]. In addition to traditional cardio-metabolic risk factors, familial and genetic factors also contribute to the development of atherosclerosis [8,9,10].

The intima-medial thickness (IMT), which is the distance between the lumen–intima and adventitia–media borders of blood vessels, is an early preclinical phenotype of atherosclerosis. Carotid IMT (cIMT) can be measured easily and noninvasively by high-resolution B-mode ultrasound with high reliability_._ It is significantly correlated with the development of atherosclerosis, as well as the risks of cardiovascular diseases (CVDs), such as myocardial infarction and stroke [11,12]. Currently, cIMT is widely recognized as a valid indicator of vascular health.

Breast cancer susceptibility gene 1 (BRCA1) binds to and recruits PALB2 (the partner and localizer of BRCA2) to the DNA damage sites, and the BRCA1–PALB2 interaction is required to activate the G2/M checkpoint upon DNA damage [13,14]. BRCA1 also forms a heterodimer with BRCA1-associated RING-domain protein 1 (BARD1) and functions as an ubiquitin ligase [15]. The BRCA1–BARD1 complex is required for homologous DNA recombination to repair DNA double-strand breaks [15]. Defects in BRCA1 are responsible for the incidences of familial breast and ovarian cancers. It had been estimated that the average cumulative risks by age 80 years in carriers of *BRCA1* germline mutation were 72% and 44% for breast cancer and ovarian cancer, respectively [16]. In addition, BRCA1 has been shown to possess cardioprotective and anti-inflammatory roles in mice [17,18]. Further, BRCA1 is constitutively expressed in human vascular ECs and SMCs [19,20]. BRCA1-overexpressing human vascular ECs and SMCs achieved protection against inflammation- and toxin-induced reactive oxygen species (ROS) by impairing TNFα-induced ROS generation in vascular ECs and inhibiting NADPH oxidase 1 (Nox1)-dependent ROS generation in vascular SMCs, respectively [19,20]. As compared to samples from adjacent plaque-free areas of human carotid artery, BRCA1 mRNA and protein levels were significantly lower in the samples from plaque regions of human atherosclerotic carotid artery [20]. These observations suggest that BRCA1 also acts as a regulator of cardio-vascular health and its deficiency is correlated with development of atherosclerosis.

Obg-like ATPase 1 (OLA1) is a translation factor-related class that is part of the Obg family and the YchF subfamily of P-loop GTPases [21]. P-loop GTPases are involved in the regulation of diverse cellular functions, including protein translation, intracellular transport, signal transduction, and cell proliferation [22,23]. Unlike other Obg family proteins, OLA1 binds and hydrolyzes ATP more efficiently than GTP [24]. OLA1 functions as a negative regulator in the cellular responses to oxidative stress in HeLa cells [25]. OLA1 binds to and protects heat shock protein 70 (HSP70) from C-terminus of Hsp70-binding protein (CHIP)-mediated ubiquitin-dependent degradation in response to heat shock stress [26]. Recently, it has been shown in mice that OLA1, via its binding to HSP70, stabilizes mitochondria SOD2 in pulmonary artery SMCs and that loss of OLA1 causes SOD2 deficiency and an increase in the protein level of X-linked inhibitor of apoptosis (XIAP), an anti-apoptotic protein, thereby increasing proliferation of pulmonary artery SMCs in mice [27]. However, OLA1 is required for proliferation of mouse embryonic fibroblasts due to its attenuation of the translation of p21, a CDK inhibitor [28]. Moreover, OLA1 promotes proliferation of hepatocellular carcinoma cells by binding with p21 and upregulating CDK2 expression [29]. Furthermore, OLA1 plays a role in promoting cell migration through regulating expression of E-cadherin, which is required for intercellular adhesion, and focal adhesion kinase (FAK) in nonvascular cells [30,31]. Previous observations have also shown that overexpression of OLA1 promotes tumor progression and causes poor survival in patients bearing hepatocellular carcinoma, colorectal cancer, endometrial cancer and lung cancer and that overexpression of OLA1 enhances drug resistance by inducing the epithelial-to-mesenchymal transition through activation of the TGF-β/Smad signaling pathway in breast cancer [29,30,32,33,34]. Furthermore, a recent study revealed that OLA1 expression is upregulated in cultured human ventricular cardiomyocytes and mouse heart after angiotensin II-induced hypertrophic response and that loss of OLA1 leads to activation of the GSK3β/β-catenin signaling pathway and, thereby, attenuation of angiotensin II-induced hypertrophic response in human ventricular cardiomyocytes [35]. However, there are no reports demonstrating the physiological role of OLA1 in human vascular ECs and SMCs.

In addition to traditional cardio-metabolic risk factors, such as high blood pressure and lipids, up to 50% of cIMT variations could be explained by genetic factors [9]. It has been shown that the methylation changes at specific CpG sites in *BRCA1* are associated with cIMT and that BRCA1 protects human vascular ECs and SMCs from oxidative stresses [19,20,36]. Further, OLA1 has been shown to bind to the amino-terminal region of BRCA1 and the carboxy-terminal region of BARD1 for centrosome regulation [37]. OLA1 plays a role in cell migration and cell proliferation and regulates the cellular responses to oxidative and heat shock stresses [25,26,27,28,29,30,31]. These observations raise the possibility that defects in or altered expression of OLA1 is involved in the development of atherosclerosis and prompted us to determine whether polymorphisms of common genetic variants in the *BRCA1*, *BARD1*, and *OLA1* genes are correlated with cIMT thickening and could be used as indicators of cardiovascular risk.

## 2. Results

### 2.1. Five SNPs in OLA1 Gene Are Significantly Correlated with cIMT

**Figure 1:**
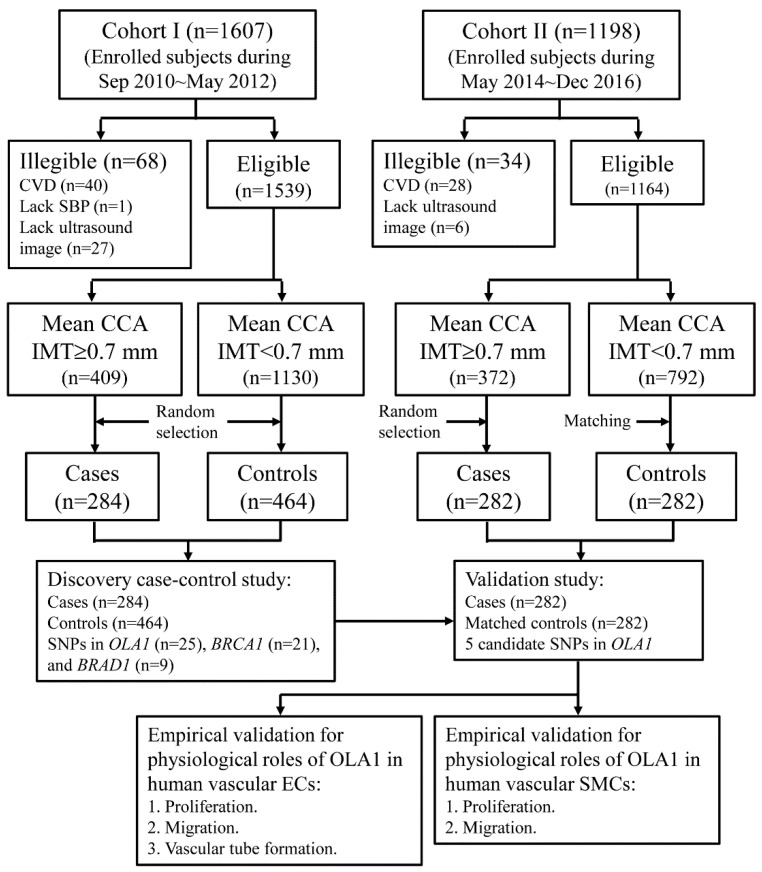
Schematic illustration of approaches to determine genetic association of *OLA1*, *BRCA1*, and *BARD1* with cIMT.

To present our approaches to determining whether *OLA1*, *BRCA1*, and *BARD1* are genetically correlated with cIMT, a schematic illustration of this study is shown in Figure 1. The anthropometric and clinical characteristics of the study subjects in the discovery and validation stages have been described previously [38]. In the discovery stage, the mean (SD) ages at enrollment for cases and controls were 58.9 (8.8) and 52.6 (8.7) years, respectively. The percentages of male participants were 55.7% and 45.9% in the case and control groups, respectively. In the validation study, the mean (SD) ages at enrollment for cases and controls were 58.5 (8.5) and 58.0 (8.5) years, respectively. The percentage of male participants in both groups was 50.7%. The detailed anthropometric and clinical profiles of the study subjects are showed in Supplementary Table S1. The results of multivariate logistic regression analyses showed that older age, male sex, higher SBP, higher BMI, higher LDL-C/HDL-C ratio, and cigarette smoking were significantly correlated with higher likelihoods of having thicker cIMT [38].

Among 59 screened SNPs, one SNP (*BARD1* rs10498020) with a *p*-value in the HWE test <0.001 and another three SNPs (*OLA1* rs13019401 and rs16862465 and *BARD1* rs34066909) with call rates <95% were excluded (Supplementary Table S2). There were 25, 21, and 9 eligible SNPs in *OLA1*, *BARD1*, and *BRCA1*, respectively, for association analyses. The pre-set critical values of the candidate genetic markers were 0.0040, 0.0048, and 0.011, respectively. A total of five SNPs in *OLA1* were eligible for the validation study. None of SNPs in *BRCA1* or *BRAD1* had a *p*-value less than the pre-set critical values of candidacy. The primers for PCR reaction and annealing of these five candidate *OLA1* SNPs are shown in Supplementary Table S3.

In the discovery case–control study, the minor allele frequencies of these five candidate *OLA1* SNPs in the controls ranged from 0.198 to 0.268 and were 0.227 to 0.310 in the cases (Table 1). The minor alleles of all candidate SNPs were significantly more prevalent in the cases. After adjustment for all significant anthropometric and clinical characteristics, heterozygous genotypes were correlated with significantly higher ORs of having thicker cIMT. The multivariate-adjusted ORs ranged from 1.59 to 1.86. Similar results were observed in the validation study.

The results of the Breslow–Day test showed that the estimated ORs for all candidate SNPs in the discovery case–control study were not statistically different from those in the validation study (all *p*-values > 0.2). Accordingly, it was appropriate to obtain the pooled ORs for each candidate SNPs. The pooled ORs of thicker cIMT ranged from 1.55 to 1.72.

**Figure 2:**
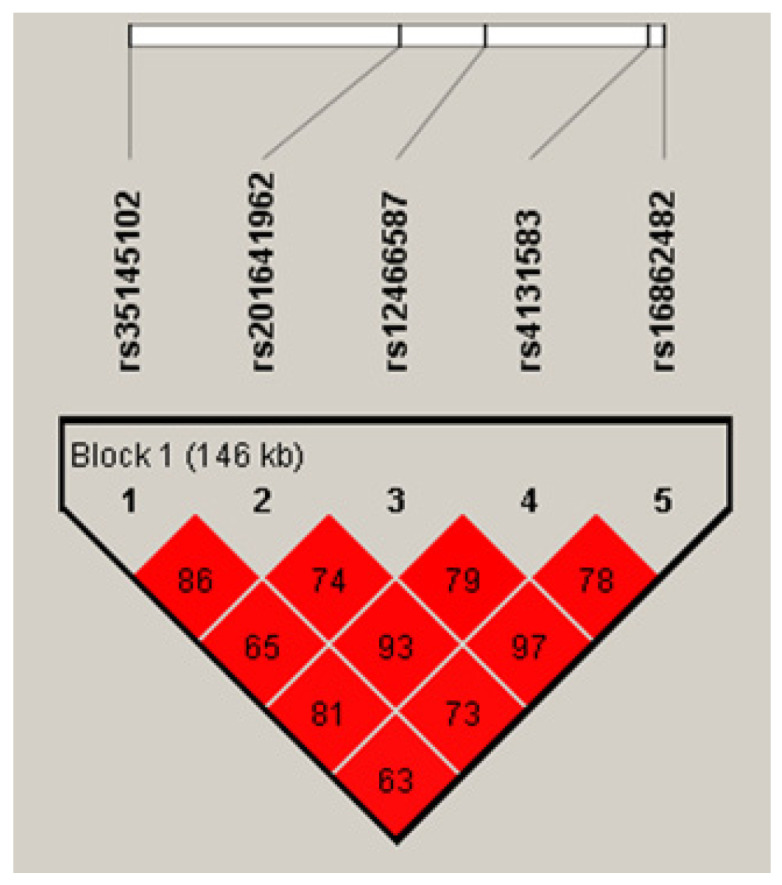
LD map of the five *OLA1* candidate SNPs (values are *r*^2^).

Linkage analyses of these five *OLA1* SNPs in the controls of the discovery case–control study showed that they formed a single LD block (Figure 2). However, SNP rs12466587 was nearly completely linked with rs16862482 (*r*^2^ = 0.97) and SNP rs35145102 showed stronger linear trends with rs201641962 and rs4131583. The most prevalent rs35145102–rs201641962–rs12466587–rs4131583–rs16862482 haplotype was H1-ATTGT, followed by haplotypes H2-GCCTC and H3-GCTTT (Table 2). The estimated ORs for these prevalent haplotypes in the discovery case–control study were not statistically different from those in the validation study (all *p*-values > 0.2). The pooled OR for H1 haplotype was significantly decreased (OR = 0.72, 95% CI: 0.59–0.86) and for the H2 haplotype it was significantly increased (OR = 1.37, 95% CI: 1.12–1.69).

The results of association analyses of multi-locus genotypes with thicker IMT are shown in Table 3. In the controls of the discovery case–control study, four multi-locus genotypes had frequencies ≥3.0%, including H1 + H1 (53.7%), H1 + H2 (27.2%), H1 + H3 (4.5%), and H2 + H2 (4.7%). The H1 + H1 genotype was negatively and the H1 + H2 genotype positively correlated with thicker cIMT. The estimated ORs for all four prevalent multi-locus genotypes were not statistically different between studies at the discovery and the validation stages (all *p*-values > 0.2). The pooled ORs of having thicker cIMT were 0.58 (95% CI: 0.46–0.75) and 1.60 (95% CI: 1.23–2.08) for the H1 + H1 and H1 + H2 genotypes, respectively.

### 2.2. OLA1-Depleted Human Aortic SMCs Are Defective in Proliferation and Migration

Vascular ECs and SMCs play the key roles in the initiation and progression of athersclerosis and the stability of vascular plaque (see reviews in references [1,2,3,4,7]). Endothelium forms a barrier between blood and tissues, and its lesion increases its permeability to and accumulation in the subendothelial matrix of apoprotein-B-containing lipoproteins (apoB LPs), which in turn leads to fibrinolysis and migration and proliferation of vascular SMCs (see reviews in references [1,2,3,4]). Based upon our observations that the common genetic variations in the *OLA1* gene we identified were statistically significantly associated with cIMT (Table 1, Table 2 and Table 3), we further investigated the physiological roles of OLA1 in human vascular SMCs and ECs, as described in this section and the subsequent section, respectively.

**Figure 3:**
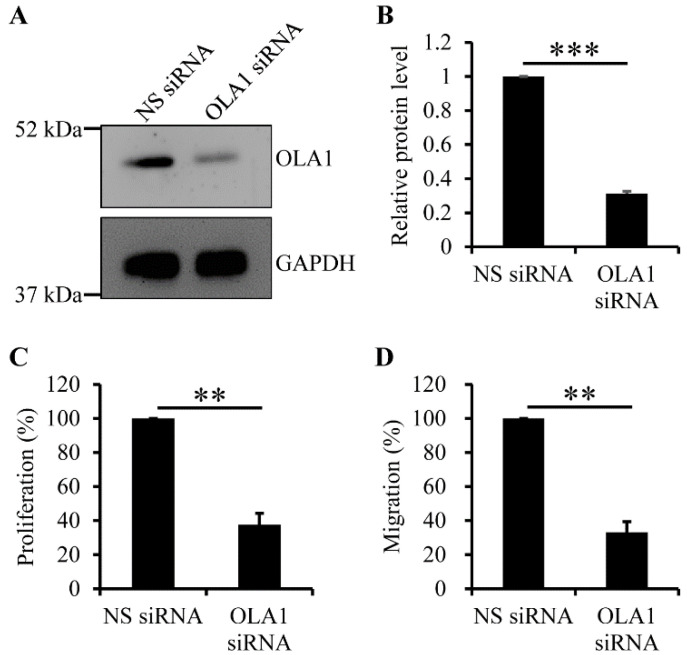
Effect of OLA1 RNAi (RNA interference) on proliferation and migration of human aortic SMCs. (**A**,**B**) Depletion of OLA1 in human aortic SMCs. After human aortic SMCs were transfected with non-silencing (NS) or OLA1 siRNA duplexes for 42 h, cells were collected and lysed for immunoblot analysis with antibodies against OLA1 and GAPDH (loading control). Immunoblot images of both OLA1 and GAPDH are shown in (**A**). The band intensities of OLA1 and GAPDH from (**A**) were determined using LS software from VisionWorks (UVP, LLC). The band intensity of OLA1 was normalized to that of GAPDH, and the relative protein levels of OLA1 normalized to the non-silencing treatment in the graph shown in (**B**) are the means ± s.d. (error bars) from three independent experiments. ***, *p* < 5 × 10^−7^. (**C**) Depletion of OLA1 causes a reduced proliferation of human aortic SMCs. After human aortic SMCs were transfected with the indicated siRNA duplexes for 42 h, 1.5 × 10^4^ cells were incubated with the BrdU-containing medium for 8 h. The BrdU-incorporated cells were stained using 3,3′-diaminobenzidine tetrahydrochloride for analysis (see the Materials and Methods section (Section 4.8)). Each independent experiment was quadruplicated. In the graphs, values are the relative percentages of the amounts of incorporated BrdU in cells normalized to the non-silencing treatment and the means ± s.d. (error bars) from three independent experiments. ***, *p* ˂ 5 × 10^−4^ (Student’s *t*-test). (**D**) Depletion of OLA1 causes reduced migration ability in human aortic SMCs. After human aortic SMCs were transfected with the indicated siRNA duplexes for 42 h, 3 × 10^4^ cells were resuspended in 100 μL of 0.5% FBS medium for the trans-well assay (see the Materials and Methods section (Section 4.8)). The cells stuck in the pores of the filter were imaged and quantitated. Each independent experiment was triplicated. In the graphs, values are the relative percentages of migrating cells normalized to the non-silencing treatment and the means ± s.d. (error bars) from three independent experiments. **, *p* ˂ 5 × 10^−5^ (Student’s *t*-test).

It is known that vascular SMCs and SMC-derived extracellular matrix accumulate in fibrous plaques (see reviews in references [1,2,4]), indicating that vascular SMCs migrate from media to intima and, thereafter, proliferate at the intima where fibrous plaques form. Furthermore, OLA1 plays a role in promoting cell migration and proliferation through regulating expression of E-cadherin, FAK, and/or p21 in nonvascular cells [28,29,30,31]. Thus, we applied the RNAi technique to determine whether OLA1 is crucial for proliferation and migration of human aortic SMCs. The OLA1 siRNA duplexes we used did efficiently deplete OLA1 in human aortic SMCs (Figure 3A,B). Using the nuclear BrdU incorporation assay (see the Materials and Methods section (Section 4.8)), we found that, compared to non-silencing treatment, depletion of OLA1 caused a reduced proliferation of human aortic SMCs (Figure 3C). Using the trans-well assay (see the Material and Methods section (Section 4.8)), we found that, compared to non-silenced human aortic SMCs, OLA1-depleted human aortic SMCs had reduced migration ability (Figure 3D). These observations indicate that OLA1 is required for migration and proliferation of human aortic SMCs.

### 2.3. OLA1-Depleted Human Aortic ECs Are Defective in Proliferation, Migration, and Tube Formation

**Figure 4:**
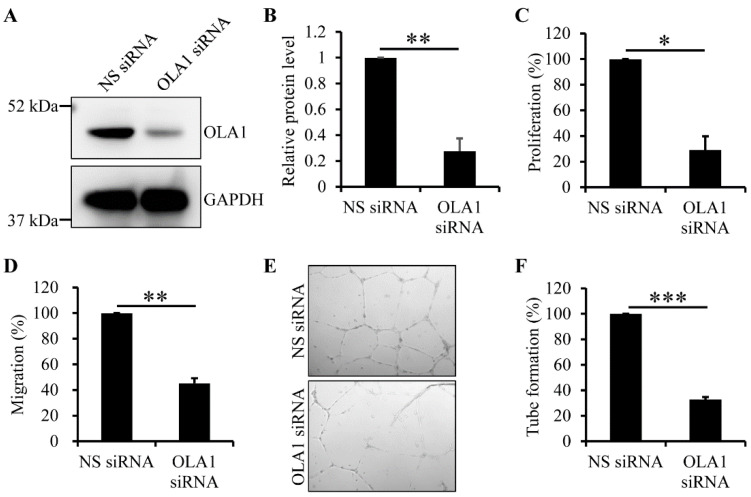
Effect of OLA1 RNAi on proliferation and migration of and tube formation by human aortic ECs. (**A**,**B**) Depletion of OLA1 in human aortic ECs. After human aortic ECs were transfected with non-silencing (NS) or OLA1 siRNA duplexes for 42 h, cells were collected and lysed for immunoblot analysis with antibodies against OLA1 and GAPDH (loading control). Immunoblot images of both OLA1 and GAPDH are shown in (**A**). The band intensities of OLA1 and GAPDH from (**A**) were determined using LS software from VisionWorks (UVP, LLC). The band intensity of OLA1 was normalized to that of GAPDH, and the relative protein levels of OLA1 normalized to the non-silencing treatment in the graph show in (**B**) are the means ± s.d. (error bars) from four independent experiments. **, *p* < 5 × 10^−5^. (**C**) Depletion of OLA1 causes a reduced proliferation of human aortic ECs. After human aortic ECs were transfected with the indicated siRNA duplexes for 42 h, 1.5 × 10^4^ cells were incubated with the BrdU-containing medium for 8 h. The BrdU-incorporated cells were stained using 3,3′-diaminobenzidine tetrahydrochloride for analysis. Each independent experiment was duplicated. In the graph, values are the relative percentages of the amounts of incorporated BrdU in cells normalized to the non-silencing treatment and the means ± s.d. (error bars) from three independent experiments. *, *p* ˂ 5 × 10^−4^ (Student’s *t*-test). (**D**) Depletion of OLA1 causes reduced migration ability in human aortic ECs. After human aortic ECs were transfected with the indicated siRNA duplexes for 42 h, 3 × 10^4^ cells were resuspended in 100 μL of 0.5% FBS medium for the trans-well assay. The cells stuck in the pores of the filter were imaged and quantitated. Each independent experiment was triplicated. In the graphs, values are the relative percentages of migrating cells normalized to the non-silencing treatment and the means ± s.d. (error bars) from three independent experiments. **, *p* ˂ 5 × 10^−5^ (Student’s *t*-test). (**E**,**F**) Depletion of OLA1 causes reduced capillary tube formation by human aortic ECs. After human aortic ECs were transfected with the indicated siRNA duplexes for 42 h, 5 × 10^4^ ECs were used for Matrigel tube formation assay (see the Materials and Methods section (Section 4.8)). The formed capillary tubes were imaged as shown in (**E**) and quantitated as shown in (**F**). Each independent experiment was triplicated. In the graph shown in (**F**), values are the relative percentages of the formed capillary tubes normalized to the non-silencing treatment and the means ± s.d. (error bars) from three independent experiments. ***, *p* ˂ 1 × 10^−6^ (Student’s *t*-test).

ECs of vasa vasorum migrate to and proliferate to form the angiogenic buds in intimal hyperplasia and atherosclerotic lesions and, after bud expansion, the angiogenic buds merge and develop new vessels, which is required for plaque growth, plaque destabilization, and thromboembolic events (see review in reference [7]). Thus, we applied the RNAi technique to determine whether OLA1 is crucial for proliferation and migration of human aortic ECs. The OLA1 siRNA duplexes we used did efficiently deplete OLA1 in human aortic ECs (Figure 4A,B). Using the nuclear BrdU incorporation assay, we found that, compared to non-silenced human aortic ECs, OLA1-depleted human aortic ECs were defective in proliferation (Figure 4C). Using the trans-well assay, we found that, compared to non-silenced human aortic ECs, OLA1-depleted human aortic ECs had reduced migration ability (Figure 4D). These observations indicate that OLA1 is required for migration and proliferation of human aortic ECs.

Given that neovascularization by ECs of vasa vasorum in atherosclerotic lesions is critical for plaque growth, plaque destabilization, and thromboembolic events (see review in reference [7]), we determined whether depletion of OLA1 may interfere with the ability of human aortic ECs to form vascular tubes and found that, compared to non-silenced human aortic ECs, OLA1-depleted human aortic ECs were defective in tube formation (Figure 4E,F). This indicates that OLA1 regulates vascular tube formation by human aortic ECs.

## 3. Discussion

In this study, we first identified five candidate SNPs—*OLA1* rs35145102, rs201641962, rs12466587, rs4131583, and rs16862482—of cIMT in a case–control study and then validated their effects in another independent frequency-matched case–control study. The relationships between these SNPs and cIMT remained statistically significant after adjustment for the effects of traditional cardio-metabolic risk factors. We also found that these five SNPs formed a single LD block and the homozygosity of the most prevalent haplotype was correlated with significantly decreased OR of thicker cIMT. Further, we found that loss of OLA1 caused reduced ability in human aortic ECs and SMCs to migrate and proliferate and reduced ability in human aortic ECs to form vascular tubes in cell culture system (Figure 3 and Figure 4).

OLA1 is ubiquitously expressed in brain, thyroid, esophagus, testis, and 23 other tissues (see information at https://www.ncbi.nlm.nih.gov/gene/29789#gene-expression) (accessed on 15 March 2021). OLA1 belongs to the YchF subfamily of P-loop GTPases, which regulate diverse cellular functions, including protein translation, intracellular transport, signal transduction, and cell proliferation [22,39]. However, to our knowledge, there are no reports on OLA1 association with preclinical traits of atherosclerosis or cardiovascular events. Further, there are no reports showing that OLA1 directly influences the functions of human immune cells and vascular ECs and SMCs. Based upon the previous observations and our observations in this study, several lines of evidence reveal that the genetic variants in OLA1 may correlate with the development of atherosclerosis.

The first line of evidence is that SNP rs35145102 is located at the 3′-untranslated region (3′-UTR) of the *OLA1* mRNA, while the others are intron variants. Given that the 3′-UTR is often targeted by microRNA for regulation of mRNA stability [40,41], we searched for the possible microRNA targeting the 3′-UTR of *OLA1* mRNA in the TargetScan database (release 7.1) at the website http://www.targetscan.org/ (accessed on 3 March 2022; see reference [42]) and found that rs35145102 resides in a possible but less conserved target sequence of hsa-miR-6770-5p at the 3′-UTR of *OLA1* mRNA (Supplementary Figure S1). This raises the possibility that rs35145102 is correlated with the expression level of OLA1. Thus, we retrieved the expression data in human immune cells by using the Ensemble Genome Browser (see the website at http://asia.ensembl.org/Homo_sapiens/Info/Index) (accessed on 15 April 2021) and found that the expression levels of OLA1 in monocytes are significantly correlated with SNPs rs35145102 and rs4131583, while those of OLA1 in macrophages are significantly correlated with SNPs rs12466587 and rs16862482 [43]. Furthermore, knockdown of OLA1 elicited an increased resistance to oxidizing agents in HeLa cells, whereas overexpression of OLA1 increased cellular sensitivity to oxidizing agents [25]. Therefore, the significant associations between these five *OLA1* SNPs and cIMT may result from the differential expression of OLA1 in immune cells, which confer the differential resistance to oxidative stresses.

The second line of evidence is that OLA1 interacts with several proteins involved in the development of atherosclerosis. Among those molecules that interact with OLA1, BRCA1 is the most promising candidate. Although none of the nine BRCA1 SNPs in this study were correlated with cIMT thickening (Supplementary Table S2), a previous study has shown that the cytosine methylation changes at specific CpG sites in *BRCA1* are associated with cIMT [36]. It is known that DNA cytosine methylation in the CpG dinucleotide usually results in gene silencing [44]. Additionally, BRCA1 mRNA and protein levels in samples of plaque-containing segments of human carotid arteries were significantly lower than those in the control samples of adjunct plaque-free segments [20]. Similarly, BRCA1 can protect human aortic smooth muscle cells against inflammation- and toxin-induced reactive oxygen species [19]. As compared with ad-null-treated *ApoE^−/−^* mice, ad-BRCA1-treated *ApoE^−/−^* mice developed significantly fewer aortic plaque lesions, exhibited reduced macrophage infiltration, and generated fewer reactive oxygen species [20]. These observations raise the possibility that a reduced expression of BRCA1 contributes to development of atherosclerosis. Further, given that OLA1 and BRCA1 regulate the cellular responses to oxidative stresses [19,20,25], it can be speculated that OLA1 and BRCA1 work together in the development of atherosclerosis.

The third line of evidence is that vascular endothelium forms a barrier between blood and tissues, and its lesion leads to fibrinolysis and migration and proliferation of vascular SMCs (see reviews in references [1,2,3,4]). Further, migration and proliferation of ECs, and then their neovascularization of vasa vasorum, in atherosclerotic lesions are crucial for plaque growth, plaque destabilization, and thromboembolic events (see review in reference [7]). In this study, we found that loss of OLA1 caused reduced ability in human aortic ECs to migrate, proliferate, and form vascular tubes (Figure 4). Therefore, it is possible that a reduced expression of or a defective mutation in OLA1 may lead to a lesion in the vascular endothelium, which occurs at the early stages of the development of atherosclerosis, or that overexpression of OLA1 promotes neovascularization by ECs of vasa vasorum, leading to plaque growth, plaque destabilization, and thromboembolic events in atherosclerotic lesions.

The last line of evidence is that it is known that one of the key steps for the development of atherosclerosis is that vascular smooth muscle cells migrate from media to intima and then proliferate in intima [2,45]. In this study, we found that OLA1 was required for migration and proliferation of human aortic SMCs (Figure 3B,C), suggesting that OLA1 should play a role in the development of atherosclerosis. However, contrary to our observation, loss of OLA1 causes an increased proliferation of mouse pulmonary artery SMCs [27]. Indeed, OLA1 is required for migration and proliferation of mouse embryonic fibroblasts [28]. Therefore, it is possible that OLA1 exerts an effect on the proliferation of cells in either a species- or cell type-dependent manner. It has been shown that OLA1 binds to and inhibits GSK3 to negatively regulate E-cadherin expression and, thereby, promotes cell migration [30]. Further, OLA1 negatively regulates cell adhesion and spreading by attenuating FAK expression and increasing cofilin phosphorylation [31]. FAK controls proliferation and migration of rat aortic SMCs, and that phosphorylation of cofilin controls platelet-derived growth factor-induced migration of human aortic SMCs [46,47,48]. Furthermore, OLA1 negatively regulates translation of p21, a CDK inhibitor, and, thereby, maintains optimal cell proliferation for developmental progression in mice [28]. Additionally, p21 can inhibit proliferation of vascular ECs and migration of vascular SMCs [49,50]. Based upon these observations, it is possible that OLA1 regulates expression of E-cadherin, FAK, and/or p21 and, thereby, controls migration and proliferation of human aortic SMCs.

To further confirm that these five *OLA1* SNPs are the major genetic determinants of cIMT, we searched for functional *OLA1* SNPs with a minor allele frequency >3.0% in the Taiwan Biobank (see the website at https://taiwanview.twbiobank.org.tw/index) (accessed on 20 April 2021). A total of six functional SNPs were identified: rs35145102, rs3739153, rs3739152, rs6737629, rs1063613, and rs10209474 (see the website at https://taiwanview.twbiobank.org.tw/index) (accessed on 20 April 2021). Except for SNP rs10209474, a 5′-UTR variant, the others are 3′-UTR variants. The LD data in the 1000 Human Genome Project Phase 3-Southern Han Chinese (see reference [51]) showed that SNPs rs3739153 and rs3739152 were in complete LD (*r*^2^ = 1.0), and these two SNPs were also in complete LD with two screened SNPs (rs12693034 and rs6741764). SNP rs6737629 was in complete LD with two screened SNPs (rs17239055 and rs2358443). SNP rs10209474 was closely linked with one screened SNP (rs12479030; *r*^2^ = 0.802). Our discovery case–control study showed that SNPs rs12693034, rs6741764, rs17239055, rs2358443, and rs12479030 were not correlated with cIMT. Accordingly, rs3739153, rs3739152, rs6737629, and rs10209474 are unlikely to play critical roles in the development of atherosclerosis. Of note, SNP rs1063613 was not included in our screened SNPs and was not linked with any other SNPs located 50 Kb down- or up-stream of its location. Its physiological significance needs further exploration.

In the study, we conducted two independent case–control studies and used two different genotyping methods to explore the relationships between common genetic variants in *OLA1*, *BRCA1*, and *BRAD1*. Additionally, we found that the minor allele frequencies of the five *OLA1* candidate SNPs in the controls were similar to the corresponding frequencies in the Taiwan Biobank, which enrolls subjects from the general population. However, there were potential limitations in the present study. The negative finding for the relationship between SNPs in *BRCA1* and *BARD1* genes and cIMT does not necessarily preclude the possibility that these two genes may play important roles in atherosclerotic diseases. In the study, only nine SNPs in *BRCA1*, in contract to 25 SNPs in *OLA1* and 21 SNPs in *BARD1*, were screened for their association with cIMT. Accordingly, *BRCA1* seems more likely to suffer from false negative bias. Additionally, to control the overall type I error and to reduce the likelihood of false negative findings at the same time, only SNPs with a *p*-value less than twofold the corrected significance level were included in the validation study. However, false-negative and false-positive findings may still exist.

There were two further potential limitations in this study. The first was that, before the start of cohort I, we conducted a prior study to evaluate the feasibility of obtaining data associated with pharmacological treatments. Most interviewees could provide the information related to the classes of medicines they took. However, many could not provide the names of medicines and the duration of medical treatment. Therefore, we did not collect detailed data associated with treatments and were unable to assess the effects of these treatments. However, based on the following reasons, our findings were more likely to be conservative. The reasons include: (a) genotypes of subjects were not influenced by treatment. (b) None of the subjects had ever received carotid ultrasonographic scans before. Therefore, it is very unlikely that individuals would take medicines to control carotid IMTs. Finally, (c) all technicians who operated the ultrasonographic systems and measured the carotid IMTs were blinded to examinees’ clinical profiles. Variations in measurements of carotid IMTs tend to be non-differential. The second limitation is that, although our study revealed that OLA1 was required to promote proliferation and migration of human aortic ECs and SMCs and vascular tube formation by human aortic ECs in a cell culture system, we cannot clarify whether loss of OLA1 in vascular ECs or SMCs would cause development of atherosclerosis or whether overexpression of OLA1 in vascular ECs or SMCs would cause development of atherosclerosis. Therefore, vascular EC- or SMC-specific inducible *OLA1* knockout mice are needed to verify whether loss of OLA1 in vascular ECs or SMCs may cause development of atherosclerosis in mice. Similarly, vascular EC- or SMC-specific inducible OLA1-overexpressing mice are also needed to verify whether overexpression of OLA1 in vascular ECs or SMCs may cause development of atherosclerosis in mice.

In conclusion, we identified five *OLA1* SNPs showing significant associations with cIMT. These genetic markers formed a closely linked LD block. The homozygosity of the most prevalent multi-locus haplotype was correlated with significantly lower ORs of having thicker cIMT. Furthermore, we found that OLA1 was required for migration and proliferation of human aortic ECs and SMCs and regulated vascular tube formation by human aortic ECs (Figure 3 and Figure 4). Given that vascular ECs and SMCs play the key roles in development of atherosclerosis [1,2,3,4,7], these genetic markers may be used for individual risk prediction of atherosclerotic diseases. These genetic markers may possess the potential for use in the development of therapeutic agents for the secondary prevention of atherosclerotic diseases.

## 4. Materials and Methods

### 4.1. Study Subjects

We used two case–control studies to screen and validate the relationships of common genetic variants in *BRCA1*, *BARD1* and *OLA1* genes with cIMT thickening. The cases and controls of the case–control studies were selected from among the participants of our community-based studies and have been described previously [38]. In brief, the subjects in the discovery stage were selected from among the participants of our previous community study (cohort I), which recruited middle-aged adults and elderly subjects from September 2010 to May 2012. The case group was a random sample of 284 subjects whose mean common carotid artery (CCA) IMT was ≥ 0.70 mm, the 75th percentile of the distribution of the mean far-wall CCA IMT. The control group was a random sample of 464 subjects who had a mean CCA IMT < 0.70 mm (Figure 1).

The study subjects of the validation study were selected from an ongoing community-based study (cohort II) that enrolled residents aged 40–74 years [38]. From May 2014 to December 2016, 1164 residents who had no CVD history and had good quality recorded carotid ultrasound images were enrolled. We selected a random sample of 282 subjects from those who had thicker cIMTs (CCA IMT ≥ 0.70 mm) as the case group. Based on the age (40–49, 50–59, and 60–74 years) and sex distributions of the case group, we performed stratified random sampling and selected 282 subjects who had normal cIMTs (CCA IMT < 0.70 mm) as the control group (Figure 1).

All participants of the two community-based studies voluntarily provided informed consent. The studies complied with the 1975 Helsinki Declaration on ethics in medical research and were reviewed and approved by the Institution Review Board of Mackay Memorial Hospital (14MMHIS075).

### 4.2. Measurements of Anthropometric and Cardio-Metabolic Profiles

The measurements of anthropometric characteristics and cardio-metabolic profiles have been described previously [52]. The anthropometric characteristics included age at enrollment, sex, body weight and height, and circumferences of the waist and hip. Blood pressure was measured three times after 10 min of rest. The cardio-metabolic profiles included systolic blood pressure (SBP), diastolic blood pressure (DBP), total cholesterol, high-density lipoprotein cholesterol (HDL-C), low-density lipoprotein cholesterol (LDL-C), fasting triglycerides (FTG), and fasting plasma glucose (FPG). A structured questionnaire was used to collect personal health behaviors, such as cigarette smoking, alcohol drinking, and dietary pattern. Personal histories of common diseases, including hypertension, hyperlipidemia, hyperglycemia, diabetes mellitus (DM), and CVDs, were also collected by the structured questionnaire.

### 4.3. The cIMT Measurement

The measurement of cIMT was performed as described previously [52]. In short, two experienced technicians who were blinded to patients’ anthropometric and clinical characteristics obtained CCA images by using high-resolution B-mode ultrasonography systems (GE Healthcare Vivid 7 and Vivid E9; General Electric Company, Milwaukee, WI, USA). A well-trained technician retrieved the digital images and measured the far-wall IMTs blindly by using automatic contouring software (GE Healthcare EchoPAC version 112.0.2; General Electric-Vingmed, Horten, Norway). The IMT was defined as the distance between the lumen–intima and media–adventitia interfaces and included plaques. The average, minimum, and maximum IMTs of the distal 1 to 2 cm of the left and right CCA were recorded. Mean cIMT was calculated as the mean of the left and right average IMTs and was used for correlation and regression analyses.

### 4.4. SNP Selection and Genotyping

In the discovery study, we used a plate (Axiom^®^ CHB 1 Array Plate; Affymetrix Ltd., Santa Clara, CA, USA) to screen the associations of common genetic variants in *OLA1, BRCA1,* and *BARD1* with cIMT. In the plate, there were 59 SNPs within 25 Kb up- or downstream of the *OLA1* (n = 27), *BARD1* (n = 23), and *BRCA1* (n = 9) genes. The eligibility of SNPs for association analyses were a call rate >95%, a *p*-value in the Hardy–Weinberg equilibrium (HWE) test in the controls that was >0.001, and a minor allele frequency >3%.

The significance level of the discovery study, designated α_corrected_, was calculated as 0.05 divided by the number of eligible SNP in the locus. To reduce the influence of over-adjustment, we used 2 × α_corrected_ as the pre-set critical value for candidate genetic markers. Eligible SNPs with a *p*-value less than the pre-set critical value for candidacy were subject to a validation study using the Sequenom iPLEX MassARRAY system (Sequenom, San Diego, CA, USA). All genotyping was performed at the National Center for Genome Medicine, Academic Sinica, Taiwan.

### 4.5. Cell Culture and Transfection

Human aortic endothelial cells (HAoEC, Promocell Inc., Heidelberg, German) were cultured in endothelial growth medium MV2 (C-22022, Promocell Inc., Heidelberg, Germany) with the addition of growth medium MV2 supplement mix (C-39226, Promocell Inc., Heidelberg, Germany), and human aortic smooth muscle cells (HAoSMC, Promocell Inc., Heidelberg, Germany) were cultured in vascular smooth muscle cell basal medium (M231500, Thermo Fisher Scientific Inc., Waltham, MA, USA) with the addition of smooth muscle growth supplement (S00725, Thermo Fisher Scientific Inc., Waltham, MA, USA). Cell transfection with siRNA duplexes was performed using Lipofectamine 3000 transfection reagent (Thermo Fisher Scientific Inc., Waltham, MA, USA) following the manufacturer’s instruction.

### 4.6. siRNA Duplexes

The OLA1 siRNA duplexes used in this study were the same as previously described [29]. The sense strand sequences of non-silencing and OLA1 siRNA duplexes were 5′-r(UUCUCCGAACGUGUCACGU)dTdT-3′ and 5′-r(GCUGCUGGAAAGUACAGAC)dTdT, respectively. The siRNA duplexes were synthesized by and purchased from GE Healthcare Dharmacon Inc., Lafayette, CO, USA.

### 4.7. Immunoblot Assay

After cells were transfected with the indicated siRNA duplexes for 42 h, cells were lysed with RIPA buffer and 200 μg cell lysates were used for the immunoblot assay. To perform the immunoblot assay, OLA1 was detected using rabbit anti-OLA1 antibodies (Abcam Inc., Cambridge, UK) and GAPDH (internal loading control) using rabbit anti-GAPDH (Genetex Inc., Irvine, CA, USA), and they were visualized by chemiluminescence (Genetex Inc., Irvine, CA, USA) and imaged with a ChemiDoc-It^®^ 815 Imager (Analytik Jena, An Endress + Hauser Company, Jena, Germany) after incubation with HRP-conjugated secondary antibodies (Genetex Inc., Irvine, CA, USA).

### 4.8. Assays for Cell Proliferation, Cell Migration, and Tube Formation

Proliferation of human aortic endothelial and smooth muscle cells was determined with the nuclear bromodeoxyuridine (BrdU) incorporation assay using a BrdU immunochemistry kit (Merk Millipore Inc., Burlington, MA, USA). The assay was performed following the manufacturer’s instruction (Millipore Inc.). In brief, cells (1.5 × 10^4^/well) were seeded on coverslips in a 24-well plate and incubated with 10 μM BrdU for 8 hours. After being washed with 1 × PBS buffer, cells were fixed with ice-cold 70% ethanol at 4 °C for 30 min. The BrdU-incorporated cells were then stained using 3,3′-diaminobenzidine tetrahydrochloride, and the stained intensity was determined by a microplate reader (Molecular Devices Inc., San Jose, CA, USA).

The migration assay for human aortic ECs and SMCs was performed using a 24-well trans-well plate with a pore size of 8 μm (Corning Inc., Corning, NY, USA). A total of 3 × 10^4^ cells were resuspended in 100 μL of 0.5% FBS medium and plated in the upper chamber, and 600 μL of 2.5% FBS medium was added into the lower chamber. After incubation at 37 °C for 4 h, cells were fixed with −20 °C methanol for 5 min and stained with 18.7 μM bisbenzimide (Sigma-Aldrich Inc., Saint Louis, MO, USA) in the dark for 15 min. After the filter was washed with 1 × PBS three times, the cells on the upper surface of filter were completely wiped away with the cotton swab. The cells stuck in the pore of the filter were imaged under a fluorescence microscope (Leica Microsystems Inc., Wetzlar, Germany) using a 40 × objective lens and the image analyzed using the QWIN software (Leica Microsystems Inc., Wetzlar, German).

A Matrigel tube formation assay was performed following the manufacturer’s instruction (BD Biosciences Inc., Mississauga, ON, Canada). The Matrigel (BD Biosciences Inc., Mississauga, ON, Canada) was dissolved at 4 °C overnight before use. Each well in the 48-well plate was coated with 150 µL Matrigel and then incubated at 37 °C for 30 min. A total of 5 × 10^4^ ECs were seeded per well on the layer of polymerized Matrigel for 24 h. The formed capillary tubes were imaged under an inverted phase contrast microscope (Zeiss Inc., Oberkochen, Germany) using a 20 × objective lens and quantified by measuring the long axis of each tube in three randomly selected fields per well using MacBiophotonics Image J software.

### 4.9. Statistical Analyses

In the study, the chi-square test was used to compare whether there were significant differences in the frequency distributions of genotypes between cases and controls. The relationships with thicker cIMT for each eligible SNP were assessed by minor allele dominant, recessive, and additive and (minor and major allele) co-dominant genotypic models and adjusted for age, sex, and other significant cardio-metabolic factors. Assessment of the pairwise LD of candidate SNPs in the control group of the discovery study was performed in Haploview 4.2 software [53].

Odds ratios (ORs) and 95% confidence intervals (CIs) were calculated with multivariate logistic regression models. The homogeneity of the estimated ORs between the discovery and validation studies was evaluated with the Breslow–Day test. The pooled ORs were obtained by summation of the study-specific ORs, weighting by the inverse of their variances when the estimated ORs were not statistically different between studies. Except for the assessment of pairwise LD, all statistical analyses were performed using SAS 9.4 (SAS Institute Inc., Cary, NC, USA).

## Figures and Tables

**Table 1:** Association analyses for five candidate SNPs with thicker carotid IMT.

	Allele B/A ^1^	Thicker cIMT		Normal cIMT		Multivariate-Adjusted		
SNP		B%	BB/AB/AA	B%	BB/AB/AA	OR ^2^	(95% CI)	P_CD_
Discovery stage								
rs35145102	G/A	31.0	17/142/125	26.8	35/179/250	1.75	(1.25–2.46)	1.1 × 10^−3^
rs201641962	C/T	28.7	14/135/135	24.2	31/163/270	1.82	(1.30–2.56)	5.6 × 10^−4^
rs12466587	C/T	22.9	10/110/164	19.8	22/139/302	1.63	(1.15–2.32)	6.3 × 10^−3^
rs4131583	T/G	28.2	14/132/138	23.1	29/156/279	1.86	(1.32–2.62)	3.6 × 10^−4^
rs16862482	C/T	22.7	10/109/165	19.9	22/141/301	1.59	(1.12–2.26)	9.4 × 10^−3^
Validation stage								
rs35145102	G/A	29.3	17/130/133	22.3	13/100/169	1.56	(1.10–2.22)	0.013
rs201641962	C/T	27.7	15/125/140	20.6	11/94/177	1.54	(1.08–2.20)	0.017
rs12466587	C/T	20.1	11/102/167	16.3	10/72/200	1.54	(1.06–2.25)	0.024
rs4131583	T/G	26.8	15/120/145	19.7	11/89/182	1.57	(1.10–2.25)	0.014
rs16862482	C/T	21.4	12/96/172	15.4	4/69/204	1.50	(1.02–2.19)	0.038
Pooled								
rs35145102	G/A	30.0	34/272/258	25.1	48/279/419	1.66	(1.30–2.12)	5.5 × 10^−5^
rs201641962	C/T	28.1	29/260/275	22.9	42/257/447	1.68	(1.31–2.15)	3.7 × 10^−5^
rs12466587	C/T	22.4	21/212/331	18.4	32/211/502	1.59	(1.23–2.06)	4.5 × 10^−4^
rs4131583	T/G	27.4	29/252/283	21.8	40/245/461	1.72	(1.34–2.20)	2.0 × 10^−5^
rs16862482	C/T	22.0	22/205/337	17.6	26/210/505	1.55	(1.20–2.00)	8.9 × 10^−4^

^1^ Alleles A and B are for major and minor alleles, respectively. ^2^ ORs were adjusted for age, sex, SBP, LDL-C, BMI, and cigarette smoking. Note: cIMT, carotid intima–media thickness; CI, confidence interval; OR, odds ratio; P_CD_: *p*-value for the co-dominant genotypic model.

**Table 2:** Association analyses for rs35145102–rs201641962–rs12466587–rs4131583–rs16862482 haplotypes with thicker carotid IMT.

		Thicker cIMT		Normal cIMT		Multivariate-Adjusted OR ^1^		
Haplotype ^2^		2n	%	2n	%	OR	(95% CI)	*p*-Value
Discovery stage								
H1	ATTGT	389	68.5	677	73.0	0.72	(0.56–0.93)	0.013
H2	GCCTC	127	22.4	182	19.6	1.30	(0.98–1.73)	0.071
H3	GCTTT	29	5.1	30	3.2	1.46	(0.83–2.57)	0.191
Validation stage								
H1	ATTGT	395	70.0	436	77.3	0.71	(0.53–0.93)	0.014
H2	GCCTC	117	20.7	84	14.9	1.49	(1.08–2.06)	0.015
H3	GCTTT	32	5.7	25	4.4	1.36	(0.78–2.36)	0.282
Pooled								
H1	ATTGT	784	69.3	1113	74.6	0.72	(0.59–0.86)	4.1 × 10^−4^
H2	GCCTC	244	21.6	266	17.8	1.37	(1.12–1.69)	2.6 × 10^−3^
H3	GCTTT	61	5.4	55	3.7	1.41	(0.95–2.09)	0.089

^1^ ORs for the presence of specific haplotypes, adjusted for age, sex, SBP, LDL-C, BMI, and cigarette smoking. ^2^ rs35145102–rs201641962–rs12466587–rs4131583–rs16862482. Note: cIMT, carotid intima–media thickness; CI, confidence interval; OR, odds ratio.

**Table 3:** Association analyses for multi-locus genotypes with thicker carotid IMT.

Multi-Locus Genotype ^2^	Thicker cIMT		Normal cIMT		Multivariate-Adjusted OR ^1^		
	n	%	n	%	OR	(95% CI)	*p*-Value
Discovery stage							
H1 + H1	123	43.3	249	53.7	0.57	(0.41–0.80)	1.1 × 10^−3^
H1 + H2	102	35.9	126	27.2	1.66	(1.16–2.37)	5.3 × 10^−3^
H1 + H3	23	8.1	21	4.5	1.60	(0.83–3.08)	0.164
H2 + H2	10	3.5	22	4.7	0.84	(0.35–2.02)	0.698
Validation stage							
H1 + H1	132	47.1	169	59.9	0.60	(0.42–0.85)	3.8 × 10^−3^
H1 + H2	89	31.8	63	22.3	1.53	(1.04–2.27)	0.033
H1 + H3	29	10.4	22	7.8	1.48	(0.81–2.69)	0.205
H2 + H2	11	3.9	9	3.2	1.46	(0.57–3.74)	0.431
Pooled							
H1 + H1	255	45.1	418	56.0	0.58	(0.46–0.75)	1.5 × 10^−5^
H1 + H2	191	33.7	189	25.3	1.60	(1.23–2.08)	4.9 × 10^−4^
H1 + H3	52	9.2	43	5.8	1.53	(0.99–2.38)	0.058
H2 + H2	21	3.7	31	4.2	1.09	(0.57–2.06)	0.80

^1^ ORs for the presence of specific genotypes, adjusted for age, sex, SBP, LDL-C, BMI, and cigarette smoking. ^2^ rs35145102–rs201641962–rs12466587–rs4131583–rs16862482. Note: cIMT, carotid intima–media thickness; CI, confidence interval; OR, odds ratio.

## Data Availability

The datasets used and/or analyzed in the current study are available from the corresponding author (Li-Yu Wang) on reasonable request.

## Supplementary Materials

The supporting information can be downloaded at: https://www.mdpi.com/article/10.3390/ijms231911511/s1.
