# Supplementary material for: Association of Common Variants in OLA1 Gene with Preclinical Atherosclerosis
Source: Int J Mol Sci. 2022 Sep 29;23(19):11511. doi: 10.3390/ijms231911511 (PMC9569939; doi:10.3390/ijms231911511)
Supplement: Supplementary file 1 [file ijms-23-11511-s001.zip › Supplementary Figure S1.pdf]

```
            OLA1:   5′ ···ACCCUUUGCCACUUAGCCUUCUCU··· 3′
                          | | |     ||  ||||||||
hsa-miR-6770-5p:   3′    AGUGCACGUUCGACACGGAAGAGU     5′
```

**Supplementary Figure S1. The possible but less conserved target site of hsa-miR-6770-5p at the 3’-UTR of *OLA1* mRNA.** The partial sequence of the 3’-UTR of *OLA1* mRNA is shown at the top and the sequence of hsa-miR-6770-5p at the bottom. The possible but less conserved target site of hsa-miRNA-6770-5p at the 3’-UTR of *OLA1* mRNA (NM_001011708.3) is shown in bold face. The nucleotide C in red in the possible hsa-miRNA-6770-5p target site is corresponding to the SNP rs35145102.
