# Supplementary material for: Association of Common Variants in OLA1 Gene with Preclinical Atherosclerosis
Source: Int J Mol Sci. 2022 Sep 29;23(19):11511. doi: 10.3390/ijms231911511 (PMC9569939; doi:10.3390/ijms231911511)
Supplement: Supplementary file 1 [file ijms-23-11511-s001.zip › Supplementary Table S1.pdf]

Supplementary Table S1. Clinical characteristics of subjects of the discovery and validation stages[1].

Variable	Discovery stage			Validation stage		
	Cases (n=284)	Controls (n=464)	p-value	Cases (n=282)	Controls (n=282)	p-value
Male sex, n (%)	157 (55.3)	213 (45.9)	0.013	143 (50.7)	143 (50.7)	1.00
Age at enrollment (years)	58.9 (8.8)	52.6 (8.7)	<0.001	58.5 (8.5)	58.0 (8.5)	0.46
Cigarette smoking, n (%)	52 (18.6)	74 (16.1)	0.40	48 (17)	38 (13.5)	0.24
BMI (kg/m^2)	25.9 (3.3)	24.1 (3.5)	<0.001	25.7 (3.2)	24.4 (3.6)	<0.001
Waist circumference (cm)	84.9 (8.4)	80.1 (9.5)	<0.001	83.8 (8.6)	81.9 (9.5)	0.011
Hip circumference (cm)	96.5 (6.2)	94.5 (9.2)	<0.001	96.4 (6.1)	94.4 (6.9)	<0.001
Waist-to-hip ratio (%)	88.0 (6.4)	84.9 (7.4)	<0.001	86.9 (6.1)	86.7 (7.1)	0.67
SBP (mm Hg)	135.0 (17.7)	126.1 (19.5)	<0.001	135.2 (18.2)	128.6 (19.5)	<0.001
DBP (mm Hg)	82.6 (13.4)	78.6 (13.3)	<0.001	82.3 (13.8)	79.7 (14.6)	0.029
Hypertension, n (%)	134 (47.4)	134 (28.9)	<0.001	128 (45.4)	93 (33.0)	0.003
Total cholesterol (mg/dL)	216.2 (42.6)	205.7 (36.0)	<0.001	213.7 (37.9)	208.9 (38.3)	0.13
LDL-C (mg/dL)	133.8 (35.8)	121.4 (33.2)	<0.001	131.6 (31.3)	123.2 (35.5)	0.029
HDL-C (mg/dL)	52.0 (14.4)	56.9 (16.0)	<0.001	53.1 (14.5)	56.5 (16.4)	0.010
Fasting plasma glucose (mg/dL)	108.7 (36.0)	97.1 (25.9)	<0.001	105.0 (32.3)	101.7 (29.2)	0.20
Diabetes mellitus, n (%)	39 (13.7)	25 (5.4)	<0.001	34 (12.1)	29 (10.3)	0.50
cIMT (mm)	0.799 (0.091)	0.599 (0.058)	<0.001	0.784 (0.087)	0.612 (0.058)	<0.001

[1]Values in the table are mean (SD), unless specified.
