# Supplementary material for: Association of Common Variants in OLA1 Gene with Preclinical Atherosclerosis
Source: Int J Mol Sci. 2022 Sep 29;23(19):11511. doi: 10.3390/ijms231911511 (PMC9569939; doi:10.3390/ijms231911511)
Supplement: Supplementary file 1 [file ijms-23-11511-s001.zip › Supplementary Table S2.pdf]

Supplementary Table S2. SNPs within 25 Kb up- and down-stream of the *Ola1 and BRCA1* genes in the discovery case-control study

			Position	Allele	Thicker cIMT (n=284)		Normal cIMT (n=464)			
Gene	SNP	CHR	GRCh37.p13	A/B	Allele A %	AA/AB/BB	Allele A %	AA/AB/BB	P_{HWE}	Call rate
OLA1	rs17237167	2	174913886	AG	85.6	207/72/5	83.6	325/126/13	8.5E-01	100.0
	rs6756828	2	174919189	TC	73.6	155/108/21	70.6	227/201/36	3.5E-01	100.0
	rs10210192	2	174924658	AG	50.5	72/143/69	51.0	113/247/104	1.6E-01	100.0
	rs1545361	2	174926751	TC	78.8	175/96/12	79.4	293/149/21	7.1E-01	99.7
	rs2358443	2	174930016	TC	92.8	244/39/1	94.6	417/44/3	1.3E-01	100.0
	rs72920503	2	174931590	AG	32.0	20/142/122	27.6	34/188/242	7.6E-01	100.0
	rs11688834	2	174933420	TC	3.9	1/20/263	4.0	1/35/426	7.5E-01	99.7
	rs117471576	2	174933674	TC	95.7	257/24/0	96.8	427/27/1	4.1E-01	98.4
	rs35145102	2	174939083	AG	69.0	125/142/17	73.2	250/179/35	7.1E-01	100.0
	rs3754812	2	174943876	TC	91.2	236/46/2	92.7	399/62/3	7.3E-01	100.0
	rs77722475	2	174971873	TC	4.6	0/26/258	3.9	0/36/428	3.8E-01	100.0
	rs17239055	2	174973726	AG	6.0	0/34/250	4.5	2/38/423	2.6E-01	99.9
	rs77040503	2	175008007	AG	2.3	0/13/271	4.1	0/38/426	3.6E-01	100.0
	rs201641962	2	175013807	TC	71.3	135/135/14	75.8	270/163/31	3.5E-01	100.0
	rs12693034	2	175014678	TC	94.9	255/29/0	96.6	434/28/2	4.3E-02	100.0
	rs13019401[1]	2	175017665	AG	53.9	74/130/54	48.3	83/236/97	5.6E-03	90.1
	rs12466587	2	175037305	TC	77.1	164/110/10	80.2	302/139/22	2.5E-01	99.9
	rs79158224	2	175040087	TG	9.7	1/52/225	8.0	2/70/389	5.4E-01	98.8
	rs6741764	2	175052632	TC	5.1	0/29/255	3.6	3/27/434	1.1E-03	100.0
	rs10930647	2	175054934	TG	93.4	239/34/1	95.7	402/38/0	3.4E-01	95.5
	rs10930652	2	175058330	CG	7.6	2/39/243	6.0	2/52/410	8.0E-01	100.0
	rs16862465[1]	2	175060459	TC	68.7	120/135/18	71.7	220/181/32	5.3E-01	94.4
	rs4131583	2	175082308	TG	28.2	14/132/138	23.1	29/156/279	2.6E-01	100.0
	rs16862482	2	175086039	TC	77.3	165/109/10	80.1	301/141/22	3.0E-01	100.0
	rs12999294	2	175093978	TC	77.6	165/109/9	80.0	299/141/22	3.1E-01	99.6

	rs76518135	2	175127372	AG	95.4	254/26/0	95.5	412/37/2	2.5E-01	97.7
	rs12479030	2	175128672	TC	73.0	143/127/13	75.8	262/173/25	6.1E-01	99.3
BARD1	rs6720448	2	215574029	TC	36.8	45/119/120	36.0	52/230/182	1.0E-01	100.0
	rs7586950	2	215574823	AC	38.6	45/127/109	39.6	67/230/163	3.3E-01	99.1
	rs7586962	2	215574857	AG	36.4	42/122/119	38.1	65/224/175	6.2E-01	99.9
	rs13405209	2	215575104	AG	43.5	55/137/92	44.5	96/220/147	4.1E-01	99.9
	rs4673890	2	215580025	TC	91.9	240/42/2	89.5	374/83/7	3.4E-01	100.0
	rs73082543	2	215589980	TC	5.6	1/30/253	7.2	3/61/400	6.9E-01	100.0
	rs280621	2	215590582	TC	94.5	254/29/1	92.8	399/61/3	6.9E-01	99.9
	rs34066909[1]	2	215599433	AC	73.4	141/98/20	76.2	254/150/28	3.6E-01	92.4
	rs80153018	2	215603597	AG	2.6	0/15/269	3.7	2/30/431	7.1E-02	99.9
	rs10498020[1]	2	215619921	TG	62.2	115/87/53	62.9	181/159/74	3.2E-04	89.4
	rs34553657	2	215620525	TC	56.3	88/144/52	51.9	127/228/109	7.3E-01	100.0
	rs1972957	2	215623084	TC	57.4	90/146/48	54.2	137/229/98	9.0E-01	100.0
	rs56830655	2	215627999	TC	42.0	46/146/91	45.9	99/228/137	8.2E-01	99.9
	rs5031009	2	215632155	TC	61.5	106/135/41	59.2	165/218/80	5.8E-01	99.6
	rs16852666	2	215633226	TC	38.6	42/135/107	41.1	82/216/164	4.6E-01	99.7
	rs548926631	2	215633303	TC	5.6	0/32/252	5.8	3/48/413	2.3E-01	100.0
	rs17487792	2	215643500	TC	17.4	5/89/190	13.1	10/102/352	4.2E-01	100.0
	rs3768709	2	215645030	TC	60.6	106/132/46	58.9	158/229/76	6.5E-01	99.9
	rs3768707	2	215645135	AG	21.3	12/96/174	17.9	17/131/313	4.8E-01	99.3
	rs6728637	2	215650033	AG	4.8	0/27/256	4.9	1/43/416	9.2E-01	99.3
	rs79755801	2	215660253	AC	95.6	259/25/0	95.2	420/43/1	9.3E-01	100.0
	rs10498023	2	215661710	TC	95.2	253/27/0	95.2	415/42/1	9.5E-01	98.7
	rs17424051	2	215688992	CG	21.1	9/101/172	17.1	16/126/321	4.1E-01	99.6
BRCA1	rs382571	17	41171481	AG	92.0	238/45/0	92.1	390/71/1	2.3E-01	99.6
	rs116889906	17	41190371	TC	8.5	3/42/239	7.1	2/62/398	8.0E-01	99.7
	rs8176273	17	41211653	AG	63.4	117/125/41	64.1	182/228/52	1.2E-01	99.6
	rs3737559	17	41234304	TC	7.7	2/40/242	9.7	2/86/376	2.1E-01	100.0

rs2070833	17	41242849	TG	27.5	23/110/151	28.0	27/206/231	3.0E-02	100.0
rs799916	17	41243190	TG	63.3	116/125/41	64.1	183/229/52	1.2E-01	99.7
rs16942	17	41244000	TC	63.6	118/125/41	64.1	183/229/52	1.2E-01	100.0
rs11653069	17	41283377	TC	36.7	41/125/116	35.8	51/229/182	9.4E-02	99.5
rs35727110	17	41290333	TC	4.8	0/27/256	6.8	2/59/403	9.2E-01	99.9

[1], rs10498020 with a p-value of HWE test <0.001 and rs13019401, rs16862465 and rs34066909 with a call rate of <95% were excluded for analysis in this study.
