# Supplementary material for: Association of Common Variants in OLA1 Gene with Preclinical Atherosclerosis
Source: Int J Mol Sci. 2022 Sep 29;23(19):11511. doi: 10.3390/ijms231911511 (PMC9569939; doi:10.3390/ijms231911511)
Supplement: Supplementary file 1 [file ijms-23-11511-s001.zip › Supplementary Table S3.pdf]

Supplementary Table S3. Details of the primers used in the polymorphism genotyping by MassArray

SNP	GRCH38.p10	Alleles	Type of variant	Primer sequences of PCR	PCR product (bp)	Tm (NN)	Annealing primer
rs35145102	174074355	G/A	utr-variant-3-prime	ACGTTGGATGAACATGGGAGAGAAAGGGAG<br>ACGTTGGATGTAATTTGGAGACCCTTTGCC	110	46	gaACCCTTTGCCACTTAG
rs201641962	174149079	T/C	intron-variant	ACGTTGGATGAGAGCCAAGCTTCTTGAAGG<br>ACGTTGGATGGACTATAGTGGGTTGAGTTG	101	46.9	AGTGGAGAGAGCACAT
rs12466587	174172577	T/C	Noncoding transcript exon variant	ACGTTGGATGAAGGGAACAAGGCAGACCAG<br>ACGTTGGATGAGAGGGTCCCTGCAGAATCA	92	56.6	cggtTCCCTGCAGAATCATGGTCTGT
rs4131583	174217580	A/G	intron-variant	ACGTTGGATGCACAGGTGTATGCATATGTC<br>ACGTTGGATGGAGGTGAAAGTGACATACAAC	102	48.6	ggGCAAAAAGTGTACAGTCAGA
rs16862482	174221311	T/C	intron-variant	ACGTTGGATGCAAGGGTTAGAAAATCACATC<br>ACGTTGGATGAGGATGATGGTAAGGTCCTG	118	45.7	gggaTCTGGTTTTGTTGTTTCTT
